# Supplementary material for: Differential Gene Expression Induced by Different TLR Agonists in A549 Lung Epithelial Cells Is Modulated by CRISPR Activation of TLR10
Source: Biomolecules. 2022 Dec 22;13(1):19. doi: 10.3390/biom13010019 (PMC9855645; doi:10.3390/biom13010019)
Supplement: Supplementary file 1 [file biomolecules-13-00019-s001.zip › biomolecules-1940708-supplementary-conversion.pdf]

Supplement: **Differential Gene Expression Induced By Different TLR Agonists In A549 Lung Epithelial Cells Is Modulated By CRISPR Activation Of TLR10**

1. sgRNA sequences and primers for amplification of the target region

1.1 Spacer sequences

Table S1. Spacer sequences

sgRNA1: caguagcaugcagaagaggg
sgRNA2: gcuaaagcccgcaagaggcu
sgRNA3: gcguggaugauucguugaca
sgRNA4: uccucagaucaauagcccca

1.2 Target region specific primers

Table S2. Target region specific primers

	Forward primer sequence	Reverse primer sequence
ACT1-vitro	gccagttggtgagcgcctgtag	ccacggcttgcactctctcagc
ACT2-vitro	agcactttgggaggccgagaca	ccatccgccctcttctgcatgc
ACT3-vitro	catgcagaagagggcggatggg	acccacgctctcaagcatctgc
ACT4-vitro	gctgagagagtgcaagccgtgg	gcagaggttaacatacgctgtgggg

2. Validation of the sgRNAs

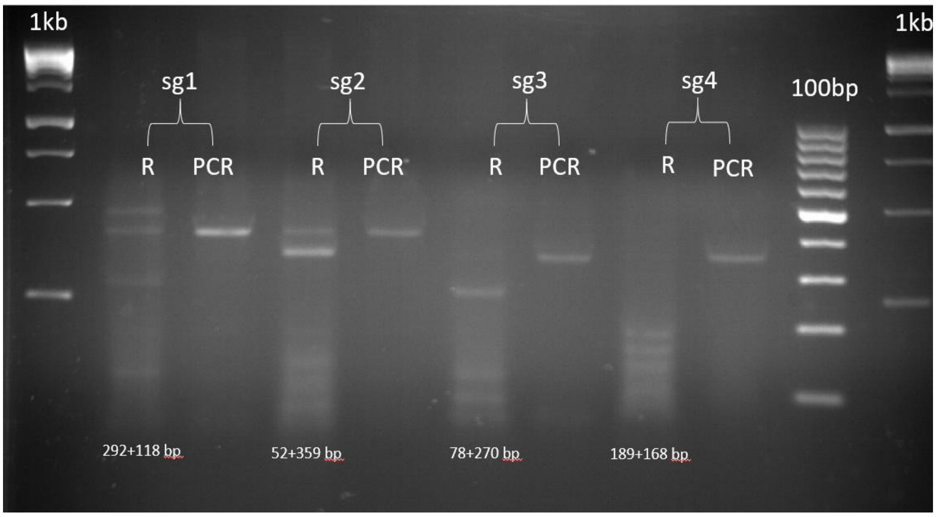

Figure S1. sgRNA validation in vitro with RNP complex. Successful binding of sgRNA and cutting of the double strand with Cas9 can be seen as digested PCR fragments.

3. Affect of overexpressed TLR10 on other membrane or cytoplasmic receptors

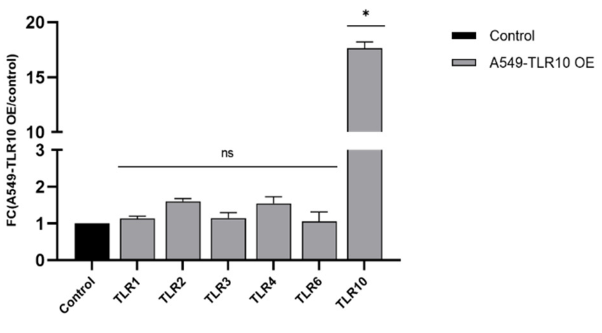

Figure S2. Effect of overexpressed TLR10 on expression of TLR2 subfamily, TLR3 and TLR4. Differential gene expression of the TLRs in A549 cells after overexpression of TLR10. Control samples were co-transfected with empty pGGa-select vector and dCas9-VPR. Data are means ± SD of three different experiments completed in triplicates, **p* < 0.05, ns= not significant.

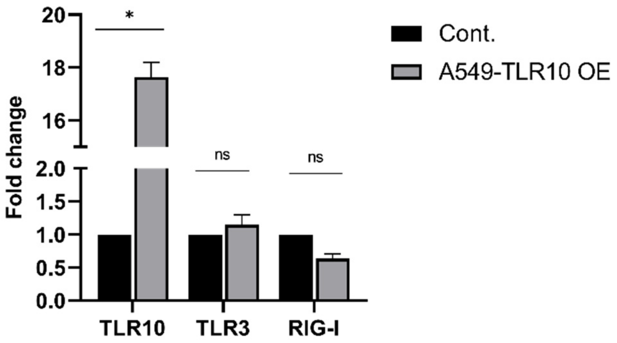

Figure S3. Basal expression of TLR3 and RIG-I. Differential gene expression of the TLR10, TLR3 and RIG-I in A549 cells after overexpression of TLR10. Control samples were co-transfected with empty pGGa-select vector and dCas9-VPR. Data are means ± SD of three different experiments completed in triplicates, **p* < 0.05.

4. Differential expression of immune associated genes in the immunostimulated A549 cells

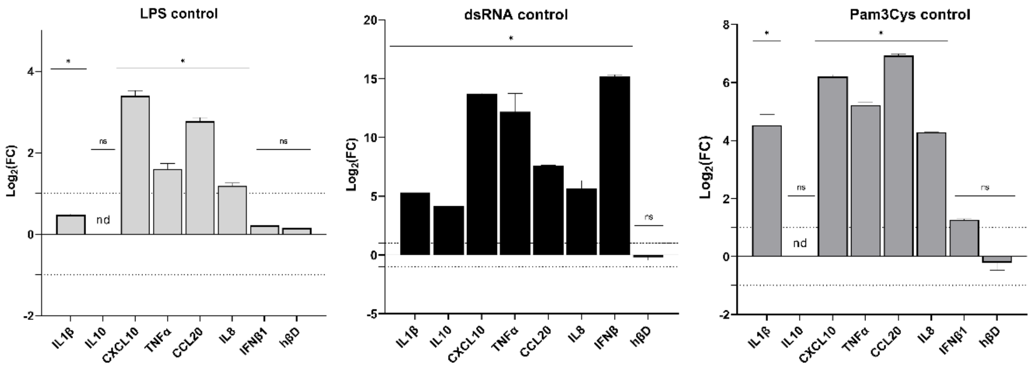

Figure S4. Differential expression of immune associated genes in the immunostimulated A549 cells. Differential gene expression (log2(FC)) of the TLRs and immune mediators in A549 cells after challenge with the different ligands, normalized to unchallenged A549 cells. Cells were stimulated with dsRNA (10 µg/ml), LPS (50 ng/ml) or Pam3Cys (50 ng/ml) for 4 hr. Data are means ± SD of three different experiments completed in triplicates, **p* < 0.05; ns= not significant, nd= not detected.

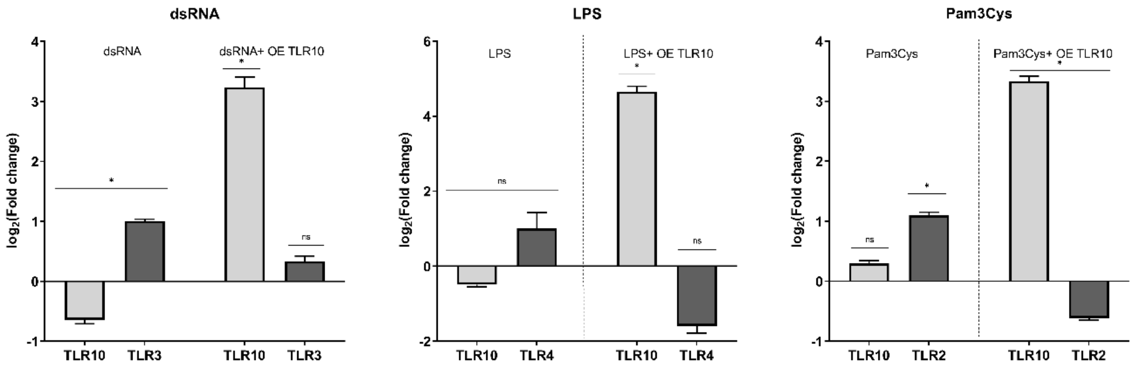

Figure S5. Differential expression of TLR10 and TLR specific for each ligand. The panel shows differential expression (log2(FC)) of TLR10 and TLR specific with each ligand, in the stimulated A549 cells and A549-TLR10 OE. Cells were stimulated with dsRNA (10 μg/ml), LPS (50 ng/ml) or Pam3Cys (50 ng/ml) for 4 hr. Data are means ± SD of three different experiments completed in triplicates, $^*p < 0.05$, ns= not significant.

5. Results from pre-designed qPCR profiling arrays

Table S3. Expression analysis of genes related to the TLR biological pathway

UniGene	GenBank	Symbol	log(A549-OE TLR10 + dsRNA)	log(A549-OE TLR10 + LPS)	log(A549-OE TLR10 + Pam3Cys)
**Hs.159494**	NM_000061	BTK	0,011	0,145	0,520
**Hs.599762**	NM_001228	CASP8	0,342	-1,065	0,230
**Hs.303649**	NM_002982	CCL2	0,612	-1,815	-0,540
**Hs.163867**	NM_000591	CD14	3,827	1,345	1,100
**Hs.87205**	NM_005582	CD180	NA	-1,435	NA
**Hs.838**	NM_005191	CD80	-0,698	1,255	-0,140
**Hs.171182**	NM_006889	CD86	NA	-0,565	-0,180
**Hs.198998**	NM_001278	CHUK	-1,580	-0,165	-0,180
**Hs.236516**	NM_014358	CLEC4E	1,792	-0,615	0,730
**Hs.1349**	NM_000758	CSF2	-6,424	0,015	-3,190
**Hs.2233**	NM_000759	CSF3	-0,657	0,725	-1,660
**Hs.632586**	NM_001565	CXCL10	3,601	-0,695	3,410
**Hs.515146**	NM_016581	ECSIT	-1,230	0,925	0,410
**Hs.131431**	NM_002759	EIF2AK2	-0,360	-1,485	-0,230
**Hs.181128**	NM_005229	ELK1	1,564	3,265	-1,050
**Hs.86131**	NM_003824	FADD	2,849	0,165	0,300
**Hs.728789**	NM_005252	FOS	0,816	-0,115	0,520
**Hs.593339**	NM_002128	HMGB1	0,345	0,755	1,000
**Hs.37003**	NM_005343	HRAS	-2,142	-0,295	0,780
**Hs.728810**	NM_005345	HSPA1A	2,299	1,765	0,000
**Hs.595053**	NM_002156	HSPD1	3,137	0,005	1,140
**Hs.37026**	NM_024013	IFNA1	-3,028	0,825	-1,820
**Hs.93177**	NM_002176	IFNB1	2,170	0,405	1,730
**Hs.856**	NM_000619	IFNG	-6,354	NA	NA

**Hs.597664**	NM_001556	IKBKB	-1,266	-0,255	-0,480
**Hs.193717**	NM_000572	IL10	-3,923	NA	1,420
**Hs.673**	NM_000882	IL12A	-1,559	-0,235	0,800
**Hs.1722**	NM_000575	IL1A	1,169	-0,655	-1,770
**Hs.126256**	NM_000576	IL1B	1,425	-1,255	-1,360
**Hs.89679**	NM_000586	IL2	NA	NA	NA
**Hs.654458**	NM_000600	IL6	-1,618	0,255	-2,940
**Hs.624**	NM_000584	IL8	1,895	-1,155	-0,280
**Hs.522819**	NM_001569	IRAK1	NA	2,595	2,810
**Hs.449207**	NM_001570	IRAK2	-0,728	0,705	-0,960
**Hs.138499**	NM_016123	IRAK4	0,007	-0,675	-0,890
**Hs.436061**	NM_002198	IRF1	-1,636	0,325	-0,310
**Hs.75254**	NM_001571	IRF3	-3,622	-0,635	0,660
**Hs.714791**	NM_002228	JUN	1,669	-0,565	-1,000
**Hs.36**	NM_000595	LTA	-0,901	0,515	-1,980
**Hs.653138**	NM_004271	LY86	-1,499	-1,255	NA
**Hs.660766**	NM_015364	LY96	-2,998	-0,975	0,740
**Hs.514012**	NM_002756	MAP2K3	-6,139	0,555	1,950
**Hs.514681**	NM_003010	MAP2K4	-1,572	-0,695	-0,270
**Hs.657756**	NM_005921	MAP3K1	-1,038	-0,985	-0,690
**Hs.644143**	NM_003188	MAP3K7	1,943	-0,525	-0,580
**Hs.431550**	NM_004834	MAP4K4	-0,656	-0,915	0,200
**Hs.138211**	NM_002750	MAPK8	-1,179	-1,475	-0,760
**Hs.207763**	NM_015133	MAPK8IP3	-0,478	0,265	-0,480
**Hs.82116**	NM_002468	MYD88	-1,199	0,295	-2,500
**Hs.654408**	NM_003998	NFKB1	-1,643	-0,465	-0,730
**Hs.73090**	NM_002502	NFKB2	-2,066	-0,195	-1,150
**Hs.81328**	NM_020529	NFKBIA	0,667	-0,635	-0,910
**Hs.2764**	NM_005007	NFKBIL1	1,232	1,505	1,170
**Hs.530539**	NM_006165	NFRKB	-0,007	0,515	-0,130
**Hs.591667**	NM_003298	NR2C2	0,287	-0,955	-0,910
**Hs.7886**	NM_020651	PELI1	2,026	-0,915	-0,190
**Hs.103110**	NM_005036	PPARA	3,199	-0,635	-0,290
**Hs.570274**	NM_003690	PRKRA	-1,827	-1,225	-0,190
**Hs.196384**	NM_000963	PTGS2	-2,485	-0,445	0,470
**Hs.631886**	NM_002908	REL	-0,684	-0,505	-0,320
**Hs.502875**	NM_021975	RELA	-1,670	-1,295	-0,970
**Hs.103755**	NM_003821	RIPK2	-0,839	-0,935	0,240
**Hs.532781**	NM_015077	SARM1	0,530	-1,285	-1,920
**Hs.501624**	NM_021805	SIGIRR	-0,426	0,435	-0,270
**Hs.507681**	NM_006116	TAB1	-0,222	-0,225	-0,770
**Hs.505874**	NM_013254	TBK1	1,128	1,545	1,740
**Hs.29344**	NM_182919	TICAM1	2,534	-2,105	-0,370
**Hs.710895**	NM_021649	TICAM2	2,191	-1,005	-0,820

**Hs.537126**	NM_001039 661	TIRAP	0,767	1,015	-0,240
**Hs.654532**	NM_003263	TLR1	1,179	-0,945	-0,260
**Hs.519033**	NM_003264	TLR2	NA	-0,255	-0,140
**Hs.657724**	NM_003265	TLR3	1,307	-0,135	-0,220
**Hs.174312**	NM_138554	TLR4	-0,674	-0,245	-3,420
**Hs.604542**	NM_003268	TLR5	-1,858	0,235	0,100
**Hs.662185**	NM_006068	TLR6	3,363	0,015	0,670
**Hs.659215**	NM_016562	TLR7	-1,051	-2,025	-0,730
**Hs.660543**	NM_138636	TLR8	NA	NA	NA
**Hs.87968**	NM_017442	TLR9	NA	3,155	0,610
**Hs.241570**	NM_000594	TNF	-1,433	-1,515	-1,800
**Hs.279594**	NM_001065	TNFRSF1A	-0,639	0,735	1,910
**Hs.368527**	NM_019009	TOLLIP	1,662	0,575	0,820
**Hs.591983**	NM_004620	TRAF6	-1,086	-0,925	-1,100
**Hs.524630**	NM_003348	UBE2N	-0,803	-0,665	-0,600

6. Primers for reverse transcription quantitative PCR (RT-qPCR)

Table S4. Table of primers for conformational analysis

Gene name	Forward primer sequence	Reverse primer sequence
CCL20	TTTGCTCCTGGCTGCTTTGA	AGCAGTCAAAGTTGCTTGCTTC
CXCL10	CCTGCAAGCCAATTTTGTCCA	TGTGTGGTCCATCCTTGGAA
hβDEF 2	TGGTGAAGCTCCCAGCCATC	ACATGTCGCACGTCTCTGAT
IFNβ	ATGACCAACAAGTGTCTCCT	CTGTCCTTGAGGCAGTATTC
IL10	GACTTTAAGGGTTACCTGGGTTG	TCACATGCGCCTTGATGTCTG
IL1β	ATGATGGCTTATTACAGTGGCAA	GTCGGAGATTCGTAGCTGGA
IL8	GAACTGAGAGTGATTGAGAGTGGA	CTCTTCAAAAACTTCTCCACAACC
RIG-I	ATGGAGGCTGCCACACTTTT	GCCATCATCCCCTTAGTAGAGC
TLR10	ACCGTGTCAACGAATCATCCA	ATGAGCTCAAAACCCCCACG
TLR1	AGTGTTTTCAAATTCAACCAGGA	AATGACAGGTCCAAGTGCTT
TLR2	GAGTTCTCCCAGTGTTTGGTGT	ACCTATCACTAGCAGGCAGG
TLR3	GTTGACTCAGGTACCCGATGA	ATACCTTGTGAAGTTGGCGG
TLR4	ATGCCAGGATGATGTCTGCC	GGGAGGTTGTCGGGGATTTT
TLR6	TCATGTTCCAAAAGACCTACCG	ACTCTGATAGAAAGCTCATGTCA
TNFα	GGCGTGGAGCTGAGAGATAAC	GGTGTGGGTGAGGAGCACAT
TOP1	CGCGCTCGTCCCTCC	AAATCCGCTTCGATCTGGGA
βACTIN	GGGACCTGACTGACTACCTC	AGCTTCTCCTTAATGTCACGC
GAPDH	GTCAGTGGTGGACCTGACCT	AGGGGTCTACATGGCAACTG
